# A Study on the Transnational Spillover Effects of Bank Risk and Sovereign Risk–From the Perspective of COVID-19 Epidemic Situation

**DOI:** 10.3389/fpubh.2022.940126

**Published:** 2022-06-24

**Authors:** Liqin Hu, Zimeng Wang, Shuiqing Hu, Wanqing Shi, Si Wang, Yi Wang

**Affiliations:** Economics and Management School, Wuhan University, Wuhan, China

**Keywords:** bank risk, sovereign risk, frequency domain VAR model, risk spillover, COVID-19

## Abstract

In recent years, the world economy and the global financial system have closely intertwined, deepened economic and financial integration via cross-border investments, financings, imports, and exports. Since banks serve as the core of a country's financial system, the risk status of banks directly affects the country's national credit and financial security. The current complexities of the international and domestic environments are increasing geopolitical risks. Moreover, there is increasing uncertainty recognition in the financial and economic development of all countries, more systemic banking risks, and sovereign risk transfer elements. In this scenario, resisting external risk input is essential to enhance risk prevention ability. Therefore, this paper adopted the VAR-based time domain and frequency model for a multi-dimensional analysis of the two perspectives of banking and sovereign risk spillover effects. The empirical results indicate that the entire sample under the static overflow effect always shows that most of the absorption is the banking sector risk, and sovereign risk is the leading risk spillover. In the frequency domain perspective, the short-term spillover effects between bank and sovereign risk are dominant. Moreover, in relation to the outbreak and continuous spread of the COVID-19 pandemic, the spillover effects are often dominated by adverse, long-term scenarios.

## Introduction

Exchanges between global economies have become closer in recent years. Moreover, the global financial system is increasingly intertwined through cross-border investments, financing, import and export trades, and continually deepening economic and financial integration. On the one hand, enhancing such financial correlations can significantly improve the allocational efficiency of financial resources on a global scale. On the other hand, these integrations can open opportunities for the worldwide spread of financial risks. Moreover, the mutual transmission mechanism of financial risks has dramatically changed with the increasing complexity of the global risk network. For instance, in the case of black-swan events such as the outbreak and continuing spread of the COVID-19 virus and the outbreak of the Russia-Ukraine war, there is an acceleration of systemic risk accumulation, the global financial market remains in a turbulent state, and the negative externalities of financial risks and cross-market spillover have become more prominent (1). In this context, the spillover of extreme financial risks can more likely evolve into a global systemic risk at the global macroeconomic level instead of mutual contagion in one country or a single market (2).

As the most central agent of the financial system, banking is a critical element in maintaining financial system stability (3, 4), securing domestic financial systems and economies, and connecting domestic and foreign economic and financial systems. To sum up, the global economy is a complex network that functions through banking systems (5). For example, most of the world's major financial institutions use SWIFT for direct data transfer and transaction clearing. However, in the Russia-Ukraine war, the U.S. announced the removal of some Russian banks from the SWIFT system, which greatly increased the burden of Russia's economic transactions with foreign countries. And as a resource-dependent country with a heavily export-dependent economy, such “financial sanctions” also caused greater damage to its economy, resulting in a spillover from banking the spillover from systemic risk to sovereign risk. However, as sovereign risks continue to accumulate and the country's fundamentals continue to deteriorate, the financial system will inevitably be affected, ultimately creating a vicious cycle of banking systemic risks and sovereign risks.

When banks support the economic development of other countries through cross-border operations, banking causes mutual risk spillovers between banking and sovereign sectors. In addition, the financial market is vulnerable to changes in market sentiment. At the same time, there is a more rapid response to–and risk spread from–external shocks, risk spillovers between financial systems and real economies, varied economic development levels of different regions, increasing perfection of financial markets, and shorter time of receiving and transmitting risks (6–9). Consequently, the following questions urgently need answers: Can a global risk spillover occur between the banking and sovereign sectors? What are the elements of this effect in different countries and markets? How do long-term and short-term effects differ? What affects the risk transmission? These problems need to be solved urgently.

As for the relationship between banking system risk and sovereign risk, scholars generally agree on their strong correlation (10–13). For instance, Gibson et al. (14) posit that sovereign default risk is a crucial factor in banking crises and that 1980–2005 data from emerging and developed countries indicate that bank sensitivity to sovereign risk increases with exposure. Moreover, when the financial situation deteriorates, the risk of default increases and, in turn, increases bank credit risk. A view similarly held by Alter and Schüler (15) and Beltratti (16). However, Bolton and Jeanne (17) posit that sovereign risk increases bank risk. Moreover, large direct rescues or explicit guarantees for banks are more likely to limit the short-term liquidity of government sectors and cause a sovereign debt crisis through the expanded model of financial intermediaries and government departments. Thus, later studies focused on bank risk and sovereign risk (18). Subsequently, Acharya et al. (19) construct a theoretical model to simulate the transmission relationship between bank risk and sovereign risk and find that sovereign bailouts of the financial sector by the sovereign lead to an increase in its sovereign risk; however, sovereign risk reinforces bank risk due to the financial sector's holding of sovereign bonds. Thus, later studies focused on bank risk and sovereign risk (18). For instance, Brunnermeier et al. (20) found that, during the European debt crisis, Greece, Italy, Spain, and other countries that hold large amounts of sovereign debt deteriorated their balance sheets of sovereign debt (11). Consequently, governments increased sovereign risk and formed a “rescue cycle.” Moreover, the credit crunch caused by bank risk weakened the economy and triggered a “real economy cycle” (21–24).

At the same time, risk transmission between bank risk and sovereign risk shows different characteristics in different conditions. One difference is external government intervention behavior. For example, Alter and Schüler (15) found that the main risk spillover between the banking and sovereign sectors changes as government intervention behavior changes. Banerjee et al. (25) also find that prior to the first Greek bailout, the sovereign and financial sectors exhibited a two-way negative feedback effect. However, since investors were already aware of the impending bailout and the two-way risk transfer was priced in after the first Greek bailout, this pattern disappeared in all subsequent bailouts, i.e., financial sector shocks lost their impact on the sovereign sector, but the strong and positive impact of sovereign default risk on its domestic financial institutions remained.

Another difference is in the internal sovereign and banking sectors. A study by Bruyckere et al. (12) found that the weaker fundamentals of the banking and sovereign sectors are more likely to receive risk spillovers. Avino and Cotter (13) found that the core European economies exhibit a dominant role in bank credit default swap spreads, while most peripheral European economies are characterized by a dominant role of their sovereign credit default swap spreads during the subprime mortgage crisis and the subsequent European sovereign debt crisis; Foglia and Angelini (26) also argue that the transmission mechanism of shocks between core and non-core banks/sovereigns is asymmetric.

Third, differences in countries can also lead to changes in bank and sovereign relations. For example, Yu (27) found that synchronized pre-crisis bank and sovereign credit default swaps spreading at the national level are minuscule. In the early stage of the banking crisis, there was a transference from the bank risk to sovereign risk due to government guarantees. However, as government bailouts increased, the national fiscal situation increasingly deteriorated. This was followed by the reverse spillover of sovereign risk to financial sectors and banks. Fratzscher and Rieth (28) also tested the causal relationship between sovereign risk and bank risk in some countries of the eurozone and found that the causal relationship between them is two-way in the eurozone as a whole, but from the results of a single country test, there is only a one-way causal relationship in some countries. Singh et al. (29) used dynamic methods to test the Granger causality between two risk measures in each country, similar conclusions are drawn.

The contribution of our study is 2-fold. First, the existing literature mainly focuses on the analysis of the relationship between bank systemic risk and sovereign risk within a single country or within the eurozone. This study is not limited to a specific region and period to explore the transmission between the two risks from a global perspective. Also, we have a detailed overview of bank risk and sovereign risk spillover with the outbreak of COVID-19 as the background, second, this paper focuses on the diversity of risk manifestations, that is, there are short-term and long-term differences in the possible effects of different shocks. Then the VAR model was established to evaluate the static and dynamic correlations of the two risk networks from different perspectives by using variance decomposition results in the time domain and frequency domain, and the network structure was constructed to find the main features of global risk networks.

To explore this scenario through an empirical evaluation, this paper uses data from 2012 to 2021 of bank systemic risks and sovereign risks in various countries worldwide. The VAR model in the time and frequency domains is used to evaluate the correlations and spillover effects of these two types of risks from two different perspectives. The empirical results show that the total static spillover effect under the full sample shows that the banking sector bears most of the risk absorption, while the sovereign sector is the main risk spillover; from the frequency perspective, the short-term effects of banks and sovereign risk occupy a dominant position.

## Materials and Methods

In the economic and financial cycle, different markets and different countries are interconnected through various channels and closely intertwined. In order to analyze the long-term and short-term characteristics of spillover effects, Baruník and Krehlík (30) proposed B-K spillover effect index. The generalized variance decomposition results are processed by Fourier transform, and the spectral representation of generalized variance decomposition is obtained. Thus, the D-Y index is improved (31, 32). By using the B-K spillover effect index, we can not only get the characteristics of relevance in the time dimension but also expand from the perspective of the frequency domain to explore the short-term and long-term effects of shocks, so as to measure the spillover effects in different situations.

### VAR Model Based on Frequency Domain

Considering a N-variate process $Y_{t=}{\left(y_{1,t},\ldots ,y_{N,t}\right)}^{\prime }$, *t* ∈ {1, 2, …, *T*}, described by the VAR(p) model:


$$\begin{array}{l} Y_{t}=\phi_{1}Y_{t-\text{1}}+\phi_{2}Y_{t-\text{2}}+\ldots +\phi_{p}Y_{t-p}+\epsilon_{t} \end{array}$$

In this paper, *Y*_*t*_ is bank/sovereign risk; Φ_1_, …, Φ_*p*_ is coefficient matrices; ϵ_*t*_ ~ (0, Σ),but Σ is a positive definite non-diagonal matrix. Generalized variance decompositions can be written as follow:


$${\left(\theta_{H}\right)}_{j,k}=\frac{\sigma_{kk}^{-1}\sum_{h=0}^{H}{\left({\left(\Psi_{h}\Sigma \right)}_{j,k}\right)}^{2}}{\sum_{h=0}^{H}{\left(\Psi_{h}\Sigma \Psi_{h}^{'}\right)}_{j,j}}H=1,2,3,\ldots$$

where σ_*kk*_ = Σ_*kk*_, ${\text{Ψ}}_{h}=\Phi {\left(L\right)}^{-\;1}$.

Transforming Ψ_*h*_ with Fourier transform:


$$\begin{array}{l} \text{Ψ}\left(e^{-i\omega }\right)=\;\underset{h}{\sum }e^{-i\omega b}{\text{Ψ}}_{h},\;\omega \in \left(-\pi ,\pi \right) \end{array}$$

Then the spectral density of *Y*_*t*_ can be defined as:


$$\begin{array}{l} S_{Y}\left(\omega \right)=\overset{\infty }{\underset{h=-\infty }{\sum }}E\left(Y_{t}{Y^{{}^{\prime }}}_{t-h}\right)e^{-i\omega h}=\text{Ψ}\left(e^{-i\omega }\right)\Sigma {\text{Ψ}}^{\prime }\left(e^{+i\omega }\right),\\ \omega \in \left(-\pi ,\pi \right) \end{array}$$

What *S*_*Y*_(ω) describes is how the variance of the *Y*_*t*_ is distributed over the frequency components ω. Also we can obtain


$$\begin{array}{l} E\left(Y_{t}{Y^{{}^{\prime }}}_{t-h}\right)=\;\overset{\pi }{\underset{-\pi }{\int }}S_{Y}\left(\omega \right)e^{i\omega h}d\omega \\ \text{Ψ}\left(e^{-i\omega }\right)=\;\underset{h}{\sum }e^{-i\omega b}{\text{Ψ}}_{h} \end{array}$$

Obviously the generalized causation spectrum over frequencies can be written as:


$$\begin{array}{l} {\left(f\left(\omega \right)\right)}_{j,k}\equiv \;\frac{\sigma_{kk}^{-1}|{\left(\text{Ψ}\left(e^{-i\omega }\right)\Sigma \right)}_{j,k}{|}^{2}}{{\left(\text{Ψ}\left(e^{-i\omega }\right)\Sigma \text{Ψ}\left(e^{+i\omega }\right)\right)}_{j,j}} \end{array}$$

As was explained above, (*f*(ω))_*j,k*_ represents the explanation proportion that the spectrum of the j-th variable changes when the k-th variable is impacted at a given frequency. Since ${\left(\text{Ψ}\left(e^{-i\omega }\right)\Sigma \text{Ψ}\left(e^{+i\omega }\right)\right)}_{j,j}\;=S_{Y_{j}}\left(\omega \right)$, (*f*(ω))_*j,k*_ only represents the relationship between different variables generated within a given frequency domain.

In order to make the result of variance decomposition in the frequency dimension correspond to the result in the time dimension,

Using the share of the variance at frequency ω of the jth variable occupying the entire frequency domain Γ_*j*_(ω) weighted by (*f*(ω))_*j,k*_, the weighting function is expressed as follow:


$$\begin{array}{l} \Gamma_{j}\left(\omega \right)=\frac{{\left(\text{Ψ}\left(e^{-i\omega }\right)\Sigma \text{Ψ}\left(e^{+i\omega }\right)\right)}_{j,j}}{\frac{1}{2\pi }\overset{\pi }{\underset{-\pi }{\int }}{\left(\text{Ψ}\left(e^{-i\lambda }\right)\Sigma \text{Ψ}\left(e^{+i\lambda }\right)\right)}_{j,j}d\lambda } \end{array}$$

Therefore, in the frequency domain dimension, When *Y*_*t*_ is wide-sense stationary, Spectral representation of the generalized variance decomposition is the weighted sum of the generalized causality equations on different frequencies, corresponding to the time dimension is the case when *H* → ∞. That is:


$${\left(\theta_{H\to \infty }\right)}_{j,k}=\frac{1}{2\pi }\int_{-\pi }^{\pi }\Gamma_{j}(\omega ){\left(f\left(\omega \right)\right)}_{j,k}d\omega$$

Combined with the real economic background, when a variable is impacted, it will bring short-term and long-term effects. So the generalized variance decomposition results corresponding to the frequency domain are (θ_*d*_)_*j,k*_ on the high and low frequency band d. That is:

On a frequency band *d* = (*a, b*):*a, b* ∈ (−π, π), *a* < *b*, the generalized variance decompositions are defined as:


$${\left(\theta_{d}\right)}_{j,k}=\frac{1}{2\pi }\int_{a}^{b}\Gamma_{j}(\omega ){\left(f\left(\omega \right)\right)}_{j,k}d\omega$$

Since the integral is linearly additive, summing over the whole interval of (−π, π) for (θ_*d*_)_*j,k*_ reduces the result of generalized variance decomposition _(θ_*H*→∞_)*j,k*_ in the time dimension.

Thus, (θ_*d*_)_*j,k*_ satisfies the following properties: For a subinterval *d*_*s*_ *in the set D* of an interval, satisfying ∩_*d*_*s*_∈*D*_*d*_*s*_ = ∅, and ∪_*d*_*s*_∈*D*_*d*_*s*_ = (−π, π), we can have:


$${\left(\theta_{H\to \infty }\right)}_{j,k}=\sum_{d_{s}\in D}{\left(\theta_{d_{s}}\right)}_{j,k}$$

### B-K Connectedness Measures

1. The directional spillovers on the frequency band d

From Equations above, on a frequency band *d* = (*a, b*):*a, b* ∈ (−π, π), *a* < *b*, the generalized variance decompositions are defined as:


$${\left(\theta_{d}\right)}_{j,k}=\frac{1}{2\pi }\int_{a}^{b}\Gamma_{j}(\omega ){\left(f\left(\omega \right)\right)}_{j,k}d\omega$$

where $\sum_{k=1}^{N}\sum_{d_{s}\in D}{\left(\theta_{d_{s}}\right)}_{j,k}=\;1$.

By standardizing (θ_*d*_)_*j,k*_, the measure of connectedness on the frequency band d can be obtained as follows:


$${\left(\tilde{\theta_{d}}\right)}_{j,k}=\frac{{\left(\theta_{d}\right)}_{j,k}}{\sum_{k}{\left(\theta_{\infty }\right)}_{j,k}}$$

_(θ_*d*_)*j, k*_ refers to the proportion that the influence on the variable j on the frequency band d occupies the whole frequency band when the variable k is impacted. That is, the size of the short-term / long-term spillover effect of variable k on variable j.

1. The Within Connectedness on the Frequency Band d

The total spillover effect index $C_{d}^{w}$ and the local spillover effect index ($C_{j\to }^{d}$and $C_{j\leftarrow }^{d}$) can be obtained through the generalized variance decomposition expression in the frequency domain. Hence, on the frequency band *d* = (*a, b*):*a, b*∈(−π, π), *a* < *b*, we can define:

The total spillovers on the frequency band d


$$C_{d}^{w}=\frac{\sum_{j,k=1,k\neq j}^{N}{\left(\tilde{\theta_{d}}\right)}_{j,k}}{\sum_{j,k=1}^{N}{\left(\tilde{\theta_{d}}\right)}_{j,k}}\times 100=100(1-\frac{Tr\left\{\tilde{\theta_{d}}\right\}}{\sum_{j,k=1}^{N}{\left(\tilde{\theta_{d}}\right)}_{j,k}})$$

The local spillovers on the frequency band d


$$\begin{array}{l} C_{j\to }^{d}=\overset{N}{\underset{k=1,k\neq j}{\sum }}{\left(\tilde{\theta_{d}}\right)}_{k,j}\times 100\\ C_{j\leftarrow }^{d}=\overset{N}{\underset{k=1,k\neq j}{\sum }}{\left(\tilde{\theta_{d}}\right)}_{j,k}\times 100 \end{array}$$

When d is taken in different intervals, the total spillover index in the time dimension can be subdivided into short-term and long-term total spillover indices.

### Data

In this paper, ΔCoVaR proposed by Adrian and Brunnermeier (33) is selected as the measure of systemic risk of banks (34–36). And the term structure of interest rate, credit spread, treasury yield change and real estate excess yield are selected as status variables to calculate ΔCoVaR. According to Bostanci and Yilmaz (37), we use a 5-year sovereign CDS spread as the main measure of sovereign risk (38–41). From the perspective of globalization, this paper analyzes the relationship between bank risk and sovereign risk. The selected research samples cover 13 emerging market countries^1^ and 10 developed economies on all continents (Table 1). So the research results are representative. Given the availability of the data, the sample time interval includes daily data from 17 October 2011 to 30 June 2021, from databases such as Wind, Datastream, Blomberg, etc. The country and its corresponding acronyms are shown in the Table 1.

**Table 1:** Countries and country acronyms.

	**Country**	**Acronym**	**Country**	**Acronym**
Asia	China	CHN	Thailand	THA
	Korea	KOR	Malaysia	MYS
	Japan^*^	JPN	Indonesia	IDN
	Philippines	PHL	Israel^*^	ISR
	Vietnam	VNM		
Europe	UK^*^	GBR	Poland	POL
	Netherlands^*^	NLD	Austria^*^	AUT
	Italy^*^	ITA	Germany^*^	GER
	Spain^*^	SPA	Russia	RUS
Americas	USA^*^	USA	Chile	CHL
	Mexico	MEX	Brazil	BRA
Oceania	Australia^*^	AUS		
Africa	South Africa	SAF		

*The ones with*

* *are the developed economies*.

## Results

### Characteristics of the Relationship Between Bank Risk and Sovereign Risk

Since both bank risk and sovereign risk are systemic risks in nature, the relationship between them will also show obvious characteristics of systemic risk. Combined with their respective characteristics, this paper first tests the correlation between bank risk and sovereign risk in each country, and then according to the actual characteristics, the specific characteristics of the relationship between them are summarized as: spillover, regionality and positive feedback.

1. Correlation

We calculate the correlation coefficient between bank risk and sovereign risk in representative countries, as shown in Table 2.

**Table 2:** Representative national bank risk and sovereign risk correlation coefficient in full sample and during a crisis.

**Country**	**Correlation coefficient** **(full sample)**	**Correlation coefficient** **(during a crisis)**	**Country**	**Correlation coefficient** **(full sample)**	**Correlation coefficient** **(during a crisis)**
China	0.26	0.62	Spain	0.28	0.39
Japan	0.35	0.36	UK	0.18	0.30
Indonesia	0.34	0.70	Italy	0.69	0.59
Russia	0.29	0.35	Brazil	0.61	0.63
USA	0.07	0.22	South Africa	0.15	0.22

It can be clearly seen from the table that there is a positive correlation between them in all countries, especially in Italy and Brazil, which are greatly affected by external shocks. It is not difficult to understand that banking as an important core sector of a country, bank risk and sovereign risk are difficult to separate from each other. Table 2 also shows that the correlation during the European debt crisis is basically higher than the average correlation of the entire sample time, such as Spain, where the correlation between bank risk and sovereign risk is 0.28 in the whole range, and 0.39 during the European debt crisis.

1. Spillover

Spillover refers to the external impact of sovereign risk and banking system risk on the economic and financial activities of our country or other countries. The root cause of spillover lies in the economic and financial relationship between countries in the world economy. At present, various countries have formed complex association networks among countries by means of trade and financial openness, and at the same time, complex financial and economic networks have been formed in the interdependent relationship between finance and the real economy within countries (42). The formation of multiple networks has laid the foundation for the outward radiation of sovereign risk and bank risk.

Take the crisis in Italy in 2018 as an example. We know that the Italian financial system is dominated by indirect financing, and the banking system occupies a core position in the economy, but the development of the Italian banking industry is extremely unstable. When events such as the Brexit vote took place in 2016, the banking system of Italy, a member of the European Union, also suffered a great impact. in the same year, the proportion of non-performing loans in the Italian banking system was as high as 14.38% of the total loans, ranking first in the euro zone countries. the banking industry is in jeopardy, and sovereign risk is also rising rapidly under the dual effects of external shocks and bank risks. At the same time, because France's debt to Italy accounts for 45.41% of its GDP, Sweden, Spain, the Netherlands and other countries account for 19.23, 18.48, and 13.49% of their GDP, respectively, which is further quickly transmitted to other euro zone countries through inter-state financial networks, resulting in an increase in sovereign risk and banks in other euro zone countries at the same time.

1. Regionality

**Figure 1:**
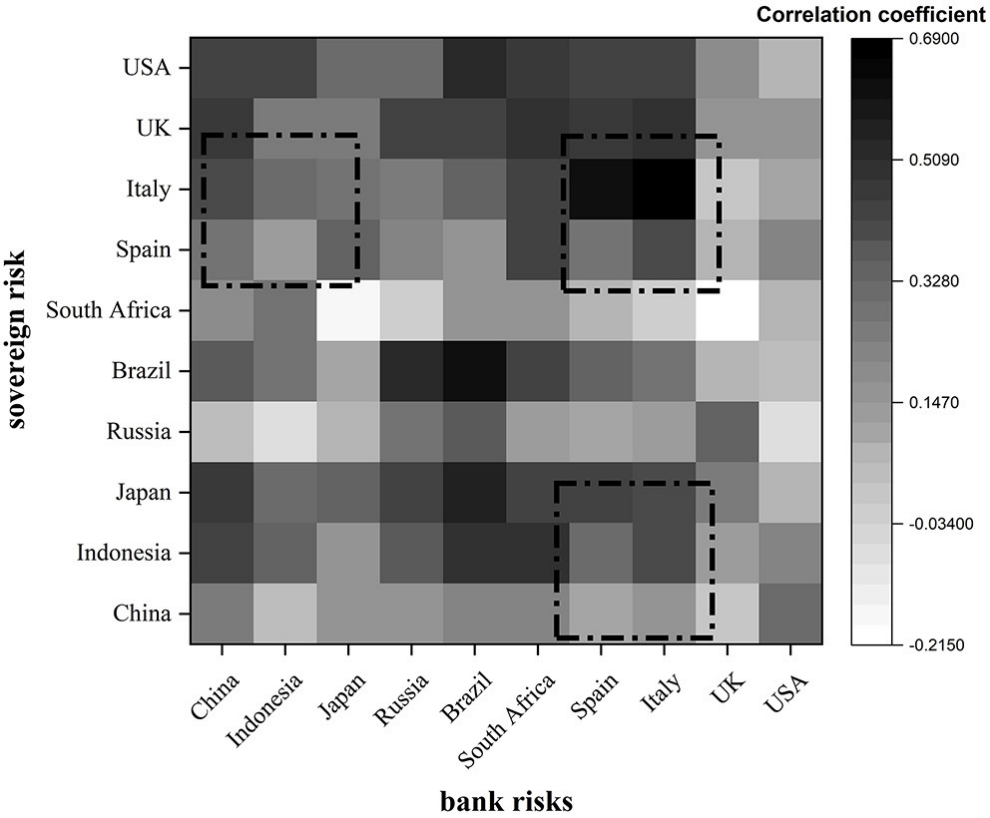
Heat map of the correlation coefficient between bank risk and sovereign risk.

As the economic, financial and trade activities within the region are closer, bank risk and sovereign risk show stronger correlation and similarity within the same region, that is, regional. Figure 1 depicts the correlation between sovereign risk and bank risk in different countries using thermal values. The darker the color in the chart, the stronger the correlation.

It is not difficult to see that the same region or countries of the same type are darker in color, that is, the correlation between banks and sovereign risk in the region is higher than that between regions. For example, the correlation coefficients between Italy and Spain, Brazil and Russia, China and Japan and Indonesia are all high. Take the darkest European region as an example, the main role of the European Union is to unify the currencies of the euro zone countries. Its birth has led to the rapid integration of the euro zone, and promoted the interaction of trade networks, economic integration and other aspects within the region. It has enhanced the economic strength of the countries in the region as a whole. With the passage of time, the economic structure of various countries began to differ greatly, and the unified implementation of monetary policy was separated from the formulation of their own fiscal policies, resulting in the gradual accumulation of fiscal deficits in some countries. The bad thing is that banks in euro zone countries are encouraged to hold a large number of sovereign debt by policies. The high concentration on such balance sheets makes the correlation between bank risk and sovereign risk within the region the most prominent.

1. Positive Feedback

Positive feedback mainly comes from the multi-round evolution of risk, which is characterized by the dynamic persistence and magnification of risk transmission. For example, if the sovereign risk of a country rises, on the one hand, it is transmitted to the sovereign sectors of other countries through international channels, and on the other hand, it is transmitted to the banking sector with the help of the relationship between financial and economic systems. When the risk reaches a certain extent, it will lead to an increase in bank risk, which in turn affects the sovereign sectors of other countries through transnational banking channels, which in turn leads to a further increase in sovereign risk. Carry out multi-round feedback and evolution in turn, cross-contagion and multi-round superposition between risks, and lower the original risk level in the accelerator effect, that is, positive feedback.

The evolution of the European debt crisis is a typical multi-round contagion superposition process. Since December 2009, when Greece announced its huge fiscal deficit and the PIIGS broke out one after another, the European debt crisis gradually escalated and triggered large-scale market panic, and most countries in the European region had their sovereign debt ratings downgraded one after another; then in the second half of 2011, the sovereign debt crisis began to spread to the banking crisis, and in September Moody's downgraded the credit ratings of two major French banks. In September, Moody's downgraded the credit ratings of two major French banks, and financial markets began to shake violently; in addition, due to the banking sector's large exposure to the sovereign debt of the “five European pigs,” the liquidity of eurozone banks fell, triggering a further deterioration of the domestic economic situation and the superposition of sovereign risks.

### Analysis of the Interval Spillover Effects in the Whole Sample

#### Static Spillover Effects Analysis of Bank-Sovereign Risk in Time and Frequency Domain

Considering the A IC and S C information criterion and sample size, we select the optimal lag order as order 1. And the introduction above shows that when the generalized variance decomposition of the time domain and frequency domain can be converted, H should be generally large enough, so we set *H* = 100. In addition, according to Baruník and Krehlík (30) definition of short term and long term, this paper defines the short term as 1–20 days and the long term as more than 20 days.

The results show that the total spillover effect index is 48.02 in the whole sample interval. Among them, the proportion of spillover index of bank risk spillover and sovereign risk spillover in time domain is 31.92 and 68.08%, respectively, and the overall spillover effect of sovereign risk is greater than that of bank systemic risk, which shows that in the global spillover network, sovereign risk is the main exporter of risk contagion. Under the frequency domain decomposition, the proportion of short-term spillover of bank risk spillover and sovereign risk spillover is 41.50 and 90.30%, respectively, which tends to be dominated by short-term effects. This may be due to the fact that markets tend to respond quickly to information and overreact more often when they are hit by negative shocks. When serious risk events occur, investor panic will spread rapidly in a short time, resulting in a sharp increase in risk spillover in the short term; but with the passage of time, investors return to rationality, so in the long run, the risk spillover effect index is small.

Generally speaking, in the cross-risk contagion between the banking sector and the sovereign sector, the sovereign sector still bears most of the risk output. In addition, no matter who is the main body of spillover between banks and sovereign risk, after comparing the proportion of long-term and short-term effects through frequency domain decomposition, we can find that short-term effects are still the main driving force in the risk network.

#### Structural Feature Analysis of Bank-Sovereign Risk Network in Time Domain and Frequency Domain

**Figure 2:**
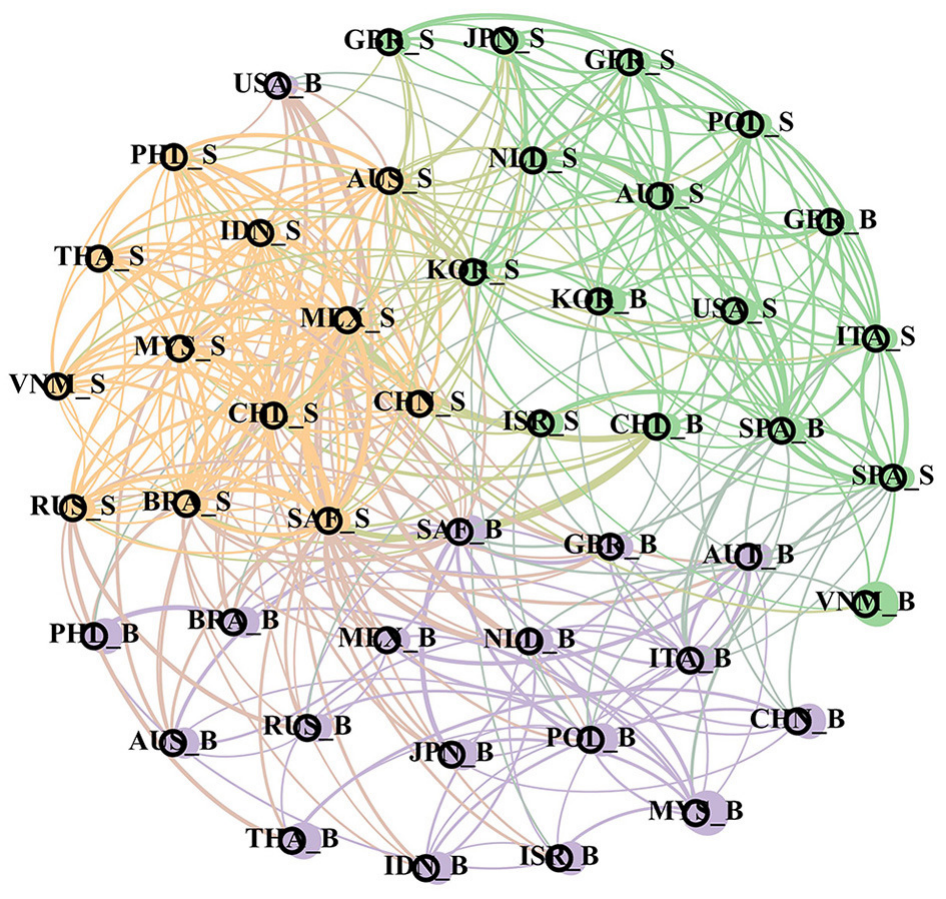
Bank-sovereign risk network in time domain.

**Figure 3:**
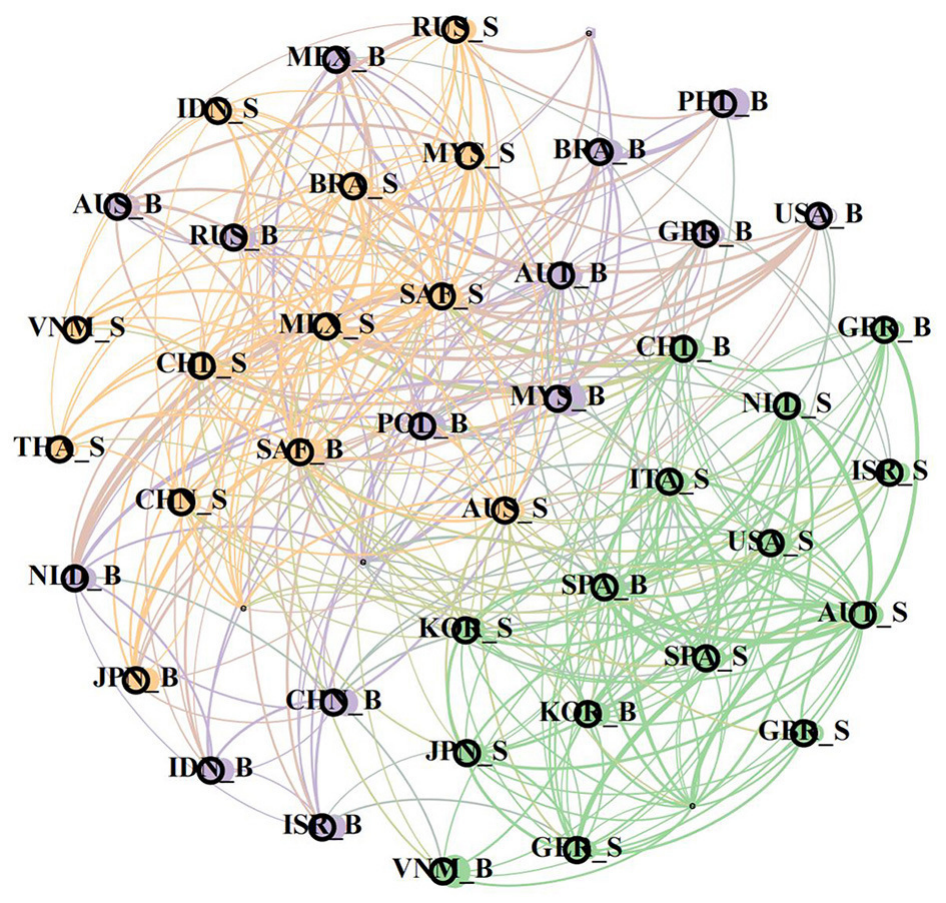
Bank-sovereign risk network in short-term.

**Figure 4:**
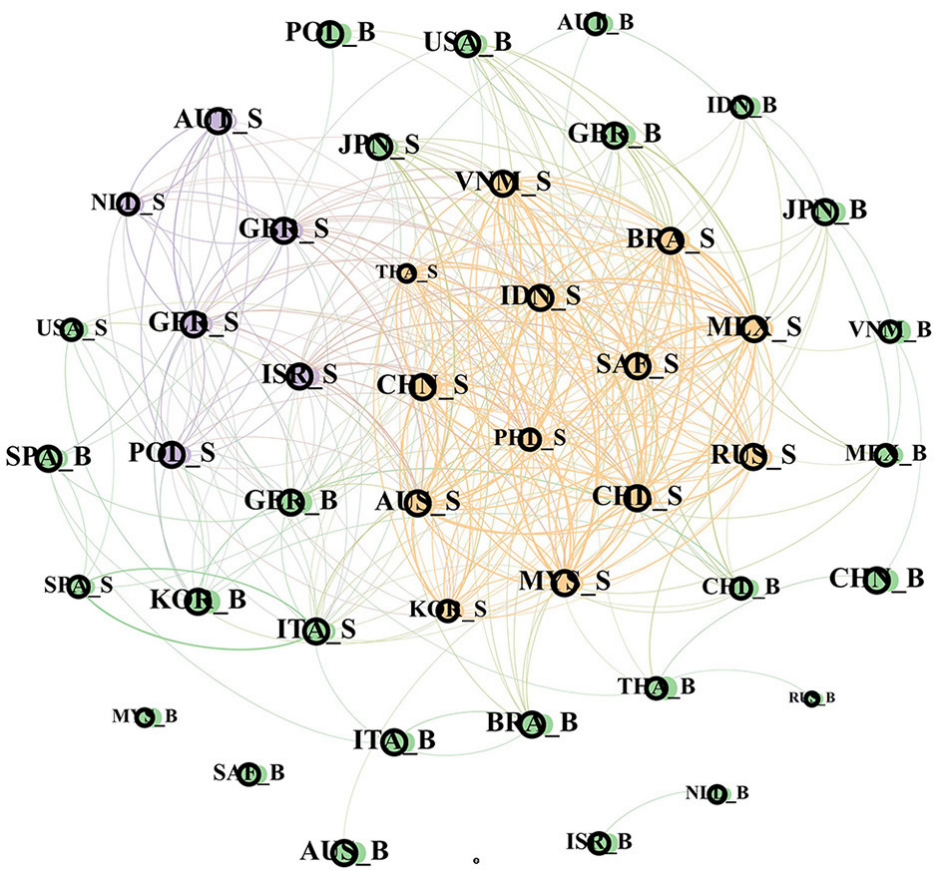
Bank-sovereign risk network in long-term.

In order to continue to capture the performance characteristics of global bank risk and sovereign risk networks in different frequency bands, this section will draw three structures: correlation network under the time domain and short-term and long-term correlation network, to further explore the transnational relationship between sovereign risk and bank risk. This article studies the sovereign risk (“_S” as the end) and bank risk (“_B” as the end) spillover effects, so there are 46 nodes in the network structure, there is a two-way overflow edge, all edges are displayed the structure is too complex, so the network will only show the top 20% of the spillover index edges, and use the “Fruchterman Reingold” algorithm to make the network structure diagram, there are greater risk spillover effects between closer nodes in the network. At the same time, in the network structure, each node will also be distinguished by its corresponding network structure characteristic index. Specifically, the node size is divided by the “degree” of each node, the greater the degree and the higher the importance of the node, divides the nodes in the whole network of each node and expresses the community modules in different colors, showing high similarity with the nodes in the community. The final time domain, short-term, and long-term sovereignty and bank risk network maps are Figures 2–**4**.

As shown in Figure 2, the modules in the global banking and sovereign risk network present three types of color division, showing significant regional clustering within each module. The orange part is composed of the sovereign risk of various emerging market countries, including China, Indonesia and Malaysia; the bank risk of most countries in the purple area is significantly larger than other modules, indicating the systemic importance of bank risk, especially in China, Indonesia, Malaysia and other countries, and the sovereign risk and bank risk of important developed economies such as the US and UK. As mentioned earlier, most of the economic and financial exchanges between countries in the world are carried out through the banking system, so the banking system of each country occupies the core position in the global banking and sovereign risk network, thus showing the clustering effects of bank risk; for the banking industry in different regions, the development differentiation in the post-crisis era has greatly changed the systematic importance of the world banking industry. For the Asian region, the rapid recovery and development from the global financial crisis have led to sustained growth in the size of banks in the past decade, while banks in Europe and other countries have been hit by the financial crisis and the European debt crisis. coupled with the tremendous changes in their financing structure, the scale is constantly shrinking, so banks in countries like China and Indonesia are becoming more and more important in the overall banking industry. Therefore, it presents a larger degree of nodes.

Figures 3, 4 dismantle (Figure 2) in the frequency domain dimension, so as to better observe the frequency domain characteristics of the network structure. By comparing the three charts, it can be found that the world risk network is still divided into three modules of different colors, and both in the time-domain risk network and in the short-term risk network and in the short-term and long-term risk network. This is because both in the short and long term, the economic and trade between different countries will tend to select the countries in the same region, so the risks within the region will show a more significant spillover effect.

Specifically, Figure 3 shows the short-term risk spillover network of banks and sovereignty. The main objects of risk spillover in the network are countries with close geographical regions and high similarity of economic fundamentals, or economic partners. It can be clearly seen that banks in most emerging market countries are closely related to sovereign risk in the short-term network, among which the sovereign risk spillover of Latin American countries such as Mexico, South Africa and Brazil is at the core. In fact, the probability of sovereign debt default of countries in Latin America is much higher than that of other regional countries, because the economic structure of these countries is relatively single, and they are all resource-dependent countries, therefore, the price fluctuations in the international commodity market will lead to severe fluctuations in sovereign risk and poor economic anti-risk ability, resulting in a large spillover of risks in Latin America in the short term.

In the long term, the regional characteristics of the network structure still remain, showing a large differentiation among different modules, so after the first 20% correlation screening, the retained part in the graph is mainly orange areas. From the previous analysis, it can be known that over time, there is a superposition effect between the transmission of bank risk and sovereign risk in each country, so the associations of different modules in the long-term risk network also change due to the inconsistent internal associations. It is worth noting that there are more connections between countries outside the orange region. Although there are still mainly countries in the region or countries with similar fundamentals, more cross-regional infections, among which, China, South Korea, the United States, the United Kingdom and other countries are in the core position. Because the several countries in the global economic and financial system in the core position, and many countries in the world have trade, financial association, and in the long term risk transmission, make the same module in the network appear more cross-regional countries, also makes China, South Korea, the United States, the UK node degree is significantly greater than other countries.

#### Dynamic Overflow Effects Analysis of Banking-Sovereign Risk in Time Field and Frequency Field

The rolling window is set to 100 in this section to calculate the total spillover index in the time dimension and the short and long-term spillover index in the frequency domain dimension, as shown in the following figure. In the figure, we can intuitively find out the dynamic evolution of the correlation degree between bank risk and sovereign risk over time, and the main drivers of the change in the total spillover effects.

**Figure 5:**
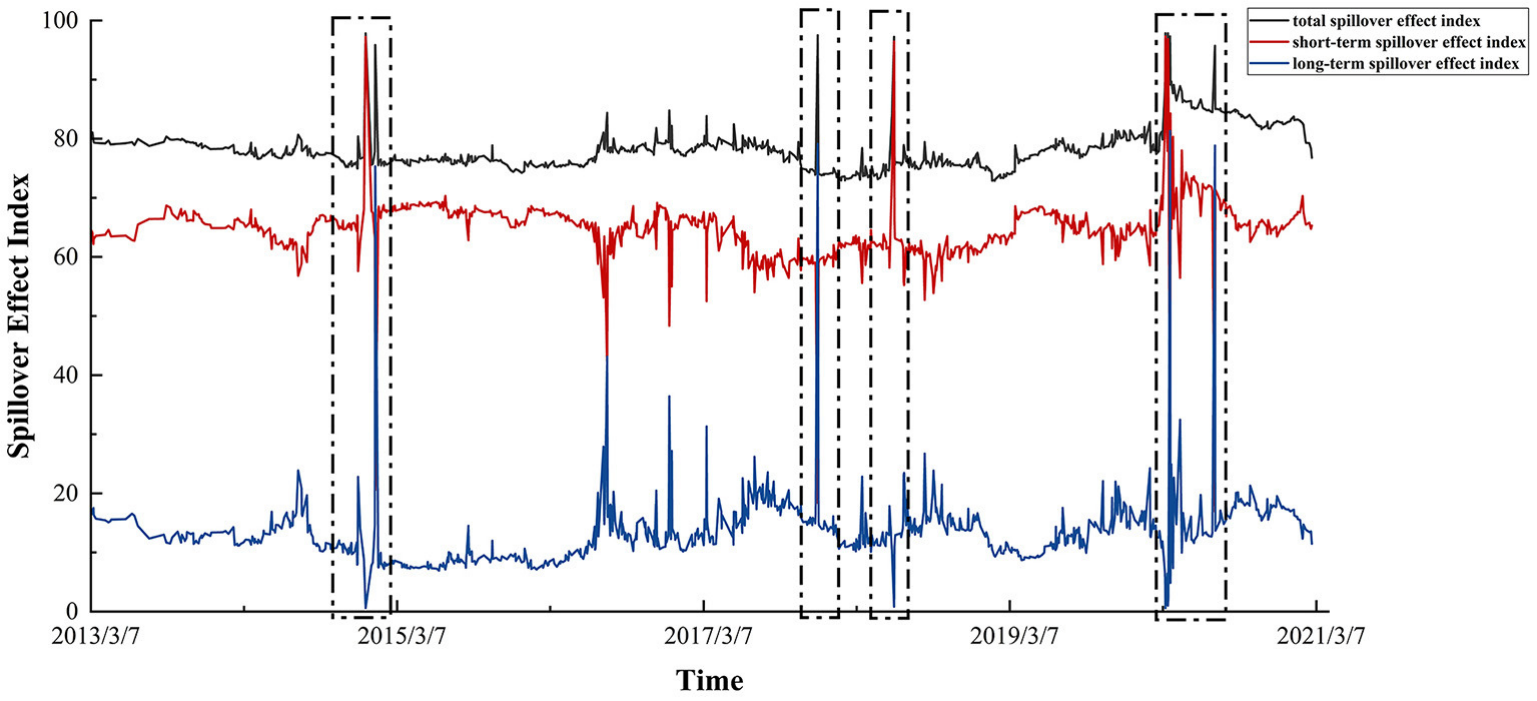
Dynamic spillover effect index of bank risk and sovereign risk.

The black line in Figure 5 shows the size of the total spillover effect index between bank risk and sovereign risk in 23 countries under the time dimension. It can be seen that the total spillover effect index has no obvious upward or downward trend since 2013, and fluctuates around 80 as a whole, indicating that there is a close risk transmission relationship between the banking industry and sovereign markets around the world. However, it can also be found that the change of the total spillover effect index shows a periodic peak, that is, it rises rapidly at several nodes, such as the beginning of 2015, the end of 2017, the middle of 2018 and the first half of 2020. Corresponding to the time interval of large fluctuations in global banking risk and sovereign risk mentioned above, it shows that the emergence of these nodes is closely related to the evolution of the global economic situation. In the event of a global crisis, the relationship between the banking sector and sovereign markets will be closer.

There are various factors in the economic cycle will have great impact on risk transmission, thus when the economic system is impacted, will produce short-term and long-term impact, the other two lines in the figure is the time dimension of total spillover effect index decomposition into short-term and long-term spillover effect index, by comparing the change of time series can explore the main driver of total spillover effects in different periods.

Therefore, the dynamic correlation between sovereign risk and bank risk can be divided into three main periods based on the changes between sovereign risk: economic recovery (2013–2015), economic shock (2016–2019) and the COVID-19 outbreak (2020-after). It can be found that (1) Under dynamic conditions, the short-term spillover effect index between bank risk and sovereign risk is still much higher than the long-term in most of the time, so the non-linear association between the two is mainly driven by the short-term effects brought by the impact. This suggests that when the market is hit, it responds quickly and lasts for a short time. However, it is worth noting that, except for a few important time nodes, because the market can process the information in time to quickly digest the impact, it will not have a lasting effect on the association between bank risk and sovereign risk, so reflected in the overall spillover effect index is the overall fluctuation around 80. (2) The changing trend of the total spillover effect index and the long-term spillover effect index is more similar. Especially when the total spillover index reaches the peak level, the long-term spillover index will also reach the peak level, so the extreme situation of the total spillover level is mainly caused by the surge of long-term spillover effects. (3) The time when the peak level of the total spillover effect index and the long-term spillover effect index appears at the same time mainly corresponds to the occurrence time of global crisis events in the real economic society, showing the characteristics of wide impact range and long duration.

### Analysis of Bank-Sovereign Risk Spillover Effects During COVID-19 Epidemic

#### Dynamic Characteristics of Bank–Sovereign Risk Spillover Effects

**Figure 6:**
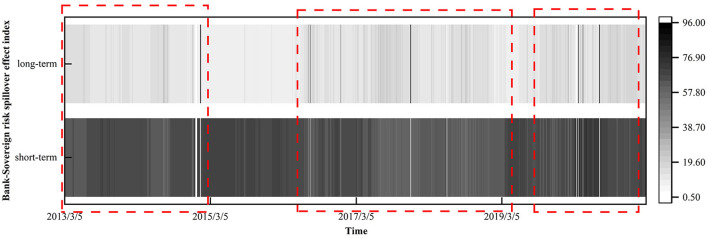
Long-term and short-term spillover index heat map of bank risk and sovereign risk.

By showing the previous dynamic spillover index in the form of a heat map as Figure 6, due to the dual impact of the global financial crisis and the European debt crisis, the long-term part of the thermal map in 2013 is darker. As the global economy gradually recovers, the overall level of long-term spillover effects is relatively low between 2014 and 2015. Although it peaked in January 2015, it quickly fell back and remained low. This shows that the relationship between sovereignty and bank risk is relatively stable during this period, although the impact of emergencies led to the long-term spread of risk around the world. But this situation did not continue, and then the economy recovered. After 2016, the long-term spillover effects between sovereign risk and bank risk was significantly higher than that in the previous period, with three consecutive highs in June 2016, December 2016 and March 2017, and reached a peak in December 2017. Among them, the fluctuation between 2016 and 2019 is more obvious, indicating that the impact of external shocks is often long-term. During this period, the overall economic instability was strengthened due to the sudden impact of commodity prices, the Federal Reserve raising interest rates and Brexit, the overall economic instability of the world, and the overall economic instability has increased. Such events lead to a rising level of sovereign risk and bank risk. at the same time, sovereign risk and bank risk are contagious through multiple channels and form a vicious circle, resulting in a lasting impact. However, due to the limited scope of some regional events and the improvement of the ability of some economies to resist risks, they have not had a significant impact around the world.

Especially after the outbreak of COVID-19 in 2020, not only the long-term spillover effect index rose in a short time, but also fluctuated more violently. Therefore, if the degree of correlation between the sovereign market and the banking industry is mainly dominated by long-term spillover effects, it shows that external shocks lead to fundamental changes in market behavior, thus affecting the overall systemic risk. At this time, the possibility of major crisis events increases. One situation is that the shock will directly affect the long-term behavior of the market, and the other is that the market cannot digest it after making a short-term response, resulting in the uncertain growth of the whole economic system and the deterioration of the economic situation. The short-term impact of the shock will be transformed into a long-term impact, forming a vicious circle between sovereign risk and bank risk, resulting in major crisis events.

The change spillover effects during the spread of the COVID-19 epidemic is more complex. Not only does the long-term spillover effect index climb in a short time, but then the spillover index fluctuates more sharply over time. The global economy was almost suspended in 2020 due to the COVID-19 outbreak. The chart shows that the relationship between sovereign risk and bank risk remains mainly affected by the short-term spillover effects between 2020 and 2021. In March 2020, the oil prices fell to negative and the influence of the global outbreak, U.S. stocks triggered four circuits, global investors' panic caused short-term capital flows, so sovereign risk and bank risk through capital flow channel transmission, causing short-term spillover effects peaked in March 2020 (43, 44). Later, the long-term spillover effect index showed an upward trend, indicating that with the spread of COVID-19, long-term economic uncertainty continued to rise, the behavior of market participants led to increased systemic risks and the relevance of global risk networks. Moreover, on the one hand, the global governments rescue (45) and introduce various policies to maintain the stability of the financial system, causing bank risk transmission through the balance sheet channel (46); on the other hand, global trade protectionism and economic shutdown prevent global trade activities, and the sovereign risk is transmitted to banks through the trade channel.

Therefore, if the degree of correlation between the sovereign market and the banking industry is mainly dominated by long-term spillover effects, it shows that external shocks lead to fundamental changes in market behavior, thus affecting the overall systemic risk, and the possibility of major crisis events increases. One situation is the impact will directly affect the long-term behavior, the other is the market short-term reaction cannot digest, leading to the whole the economic system uncertainty growth, economic situation deteriorated, the short-term impact will be transformed into long-term impact, between the sovereign risk and bank risk, major crisis events.

#### Analysis on the Change of Bank–Sovereign Risk Network Structure

In order to discuss the spillover situation of bank-sovereign risks during the COVID-19 spread period, this section focuses on the network structure characteristics during the global spread of COVID-19.^2^ Due to the complex relationship between banks and sovereign risks in the time domain, this section only retains the correlation relationship in the top 10% when constructing the network structure, so as to more clearly show the characteristics of the network structure.

It can be found in the figure that regional aggregation is still the most prominent feature of the network structure. Compared with the previous whole sample range, the period of COVID-19 mainly shows a significant increase in risk infection association among countries in the world, and risk spillover occurs among more countries in the network structure.

**Figure 7:**
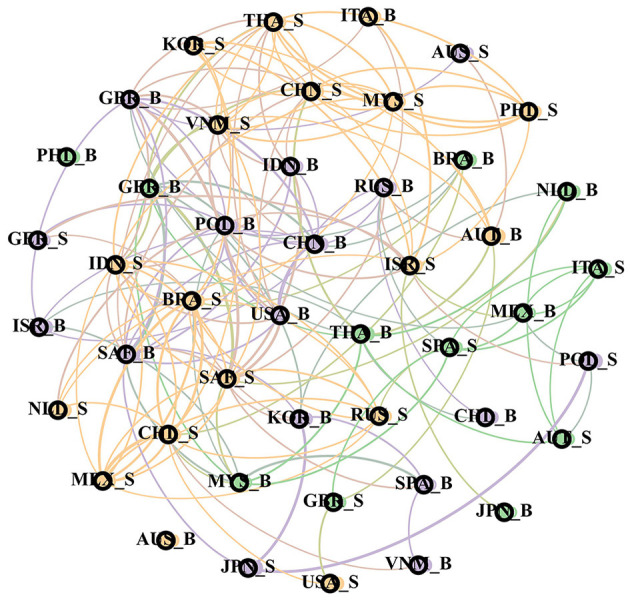
Risk network structure during the global spread of COVID-19 epidemic.

In the period of the global spread of COVID-19, it can be found that the number of edges connected by the network structure increased significantly compared with the previous two periods, indicating that the impact of the global spread of COVID-19 increases the range of risk transmission among countries around the world. In fact, such major public emergencies have brought huge challenges to the global economic development, with the internal and external macroeconomic uncertainties rising sharply, and the financial markets also showing sharp fluctuations during this period. In March 2020, US stocks suffered four circuit breakers in 10 days, stock markets in other countries plunged, financial market instability increased. More countries were affected by COVID-19. The world economy began recession. Great external uncertainty and global economic shutdown pressure further panic and global liquidity tightening. At the same time, expansionary fiscal policy became their choice to stabilize markets, sending the government deficit up sharply, with the average global public debt reaching 97% of GDP in 2020. Therefore, the sovereign risk and bank risk of many countries in the world also increase, and the complexity of the risk network in Figure 7 is also further increased.

To sum up, it can be found that the occurrence of major external events will have a significant impact on the structure of banks and sovereign risk networks. On the one hand, it will make the correlation of the global risk network increase, and on the other hand, it will change the network module through the change in economic and trade relationships.

## Discussion

From data on risk and sovereign risk of banks around the world from 2012 to 2021, this paper establishes a VAR model, evaluates the static and dynamic correlations using the variance decomposition results in the time and frequency domains, and constructs a network structure to identify the main characteristics of the global risk network. Finally, the spreading period of the COVID-19 pandemic is discussed. This study found that: First, in bank-sovereign risk infection, the banking sector bears most of the risk absorption while the sovereign sector is the primary agent of risk spillover. However, the time domain spillover effects and frequency domain decomposition indicate that, in the spillover between banks and sovereign risk, the impact of the short-term effects have consistently been large in the dominant risk network. Consequently, the market's negative impacts are affected by increasingly rapid response to information, and more time will show overreaction. Second, the transnational network spillover structure of sovereign and bank risks includes three modules in the time and frequency domains. Moreover, the transmission characteristics of different periods differ. For instance, in the short term, the regional and sectoral aggregation effect is more pronounced, such as in the Belt and Road countries in Europe and Asia. However, in the long term, the regional characteristics of the network structure remain, and the spread area of risk is larger and more apparent. Third, in the dynamic correlations between sovereign risk and bank risk in non-crisis periods, risk infection is caused by market sentiment volatility and herd behavior, which do not have lasting effects. Moreover, when major external events similar to the global spread of the COVID-19 pandemic occur, long-term spillover is dominant, indicating that the risk spreads globally through trade channels and that the fundamental differences among different countries begin to deteriorate.

Several policy implications can be drawn from our findings. First, we should establish a sound risk prevention and control mechanism. Internally, the management of cross-border capital flows should be strengthened to avoid short-term effects between bank risks and sovereign risks caused by large-scale hot capital changes, which will harm the macroeconomy. When assessing the risk of outbound investment, attention should be paid to the use of network ideas, actively identifying and preventing bank risks and sovereign risks, and establishing a good risk control system. Second, implement differentiated regulatory measures for different bank-sovereign risk transmission mechanisms to strengthen the prevention of vicious mistakes between bank risks and sovereign risks. For capital flow channels with obvious short-term effects, real-time supervision and control are required to avoid short-term market fluctuations evolving into long-term effects; for long-term balance sheet channels and trade channels, on the one hand, strengthen domestic demand construction through internal circulation, increase economic resilience and reduce the adverse effects of international uncertainty such as geopolitical turbulence and frequent trade friction.

## Data Availability Statement

The original contributions presented in the study are included in the article/supplementary material, further inquiries can be directed to the corresponding author/s.

## Author Contributions

LH conceived the idea of the study. ZW and SH designed the study, performed the research, analyzed data, and wrote the paper. WS assisted in data collection, participate in data processing, and the production of figures and tables. SW and YW were mainly responsible for organizing the paper format and material collection. All authors contributed to the article and approved the submitted version.

## Funding

This article was supported by the Major Program of the National Social Science Foundation of China (Nos. 20&ZD105 and 21ZDA114).

## Conflict of Interest

The authors declare that the research was conducted in the absence of any commercial or financial relationships that could be construed as a potential conflict of interest.

## Publisher's Note

All claims expressed in this article are solely those of the authors and do not necessarily represent those of their affiliated organizations, or those of the publisher, the editors and the reviewers. Any product that may be evaluated in this article, or claim that may be made by its manufacturer, is not guaranteed or endorsed by the publisher.

## Footnotes

^1^The MSCI Index defines the following countries as emerging market countries: Brazil, Chile, Colombia, Mexico, Peru, Czech Republic, Egypt, Greece, Hungary, Poland, cartel, Russia, South Africa, Turkey, Arab, United Arab Emirates, China, India, Indonesia, South Korea, Malaysia, Pakistan, Philippines, Thailand. According to the availability of the data, we selected Brazil, Chile, China, Indonesia, South Korea, Malaysia, Mexico, the Philippines, Poland, Russia, South Africa, Thailand, Vietnam.

^2^The global spread period of COVID-19 was selected on January 30 2020, when the WHO declared COVID-19 as a “public health emergency of international concern”, which lasted until the end of this sample on June 30, 2021.
